# Association of the fibrosis-4 index with prevalent diabetes among adults: a cross-sectional analysis of NHANES 2003–2018 and a Chinese hospital-based health examination population

**DOI:** 10.3389/fendo.2026.1881759

**Published:** 2026-07-30

**Authors:** Zeng Chen, Xiaoqing Chen, Jianqiang Wang, Sheng Hu

**Affiliations:** 1 College of Sports Medicine, Wuhan Sports University, Wuhan, China; 2 Department of Rehabilitation Medicine, Geriatric Hospital of Hainan, Hainan, China

**Keywords:** age confounding, cross-sectional study, diabetes, fibrosis-4 index, model performance, NHANES

## Abstract

**Background:**

The fibrosis-4 (FIB-4) index incorporates age, aminotransferases, and platelet count and is widely used for liver fibrosis risk stratification. Because age is part of its formula and strongly associated with diabetes, apparent associations between FIB-4 and prevalent diabetes may reflect age-related confounding rather than an independent hepatic signal.

**Methods:**

We conducted a cross-sectional analysis of 35,213 adults from NHANES 2003–2018 and 32,456 adults from a hospital-based health examination population in Hainan, China (2022-2025). Survey-weighted and conventional multivariable logistic regression evaluated FIB-4 continuously and by tertiles. Additional analyses assessed nonlinear associations, age parameterization, collinearity, liver-related exclusions, age-free liver indices, and incremental model performance.

**Results:**

In age-group-adjusted NHANES models, each 1-unit increase in FIB-4 was associated with higher odds of prevalent diabetes (OR, 1.13; 95% CI, 1.06-1.22). After replacing age categories with continuous age, this association was attenuated and no longer significant (OR, 1.03; 95% CI, 0.95-1.12; *P* = 0.518). In the hospital-based population, where Model 3 already used continuous age, the association persisted (OR, 1.36; 95% CI, 1.25-1.48). Collinearity diagnostics did not indicate problematic multicollinearity. Adding FIB-4 to traditional diabetes predictors did not improve NHANES discrimination (AUC, 0.822 vs 0.822) and produced only minimal improvement in the hospital-based population (AUC, 0.756 vs 0.758).

**Conclusions:**

The association between FIB-4 and prevalent diabetes was sensitive to age modeling and differed across datasets. FIB-4 provided little incremental model performance beyond traditional diabetes predictors. These cross-sectional findings do not support FIB-4 as an independent screening, diagnostic, or predictive tool for diabetes.

## 1 Background

Diabetes is a major chronic metabolic disease and an important contributor to global morbidity. Its pathogenesis involves insulin resistance, dysregulated lipid metabolism, chronic low-grade inflammation, and interactions between hepatic and systemic metabolic pathways (1, 2). The liver is central to glucose and lipid metabolism, and liver-related biomarkers are frequently examined in metabolic epidemiology because they may reflect hepatic injury, steatosis, inflammation, or broader cardiometabolic comorbidity (3, 4).

The fibrosis-4 (FIB-4) index is derived from age, aspartate aminotransferase (AST), alanine aminotransferase (ALT), and platelet count and was originally developed to estimate fibrosis stage in chronic liver disease (5–7). Prior studies have mainly evaluated FIB-4 among individuals with established diabetes, metabolic dysfunction-associated steatotic liver disease, or chronic liver disease (8–10, 15, 16, 24–32, 34, 41). Fewer studies have examined the association between FIB-4 and prevalent diabetes in broader adult populations while explicitly addressing the mathematical contribution of age to the FIB-4 formula. This issue is important because age is one of the strongest correlates of diabetes and may create biological and statistical circularity if not handled carefully.

The present study therefore evaluated the association between FIB-4 and prevalent diabetes in NHANES 2003–2018 and in an independent Chinese hospital-based health examination population from 2022 to 2025. The objective was not to propose FIB-4 as a diabetes prediction model, but to characterize the shape of the association, assess reproducibility in a second adult dataset, and quantify whether FIB-4 added meaningful information beyond age and traditional diabetes risk factors. FIB-4 was interpreted as an accessible composite liver-related index rather than as a causal biomarker or a validated diabetes screening tool.

## 2 Methods

### 2.1 Study design and population

This cross-sectional study analyzed data from two adult populations. The first population included participants from eight consecutive NHANES cycles conducted between 2003 and 2018. NHANES uses a complex, multistage, stratified probability sampling design to generate nationally representative estimates for the noninstitutionalized civilian population of the United States. After the survey cycles were combined and eligibility criteria based on age, FIB-4 availability, diabetes status, and covariate completeness were applied, 35,213 adults were included in the final NHANES analysis.

**Figure 1:**
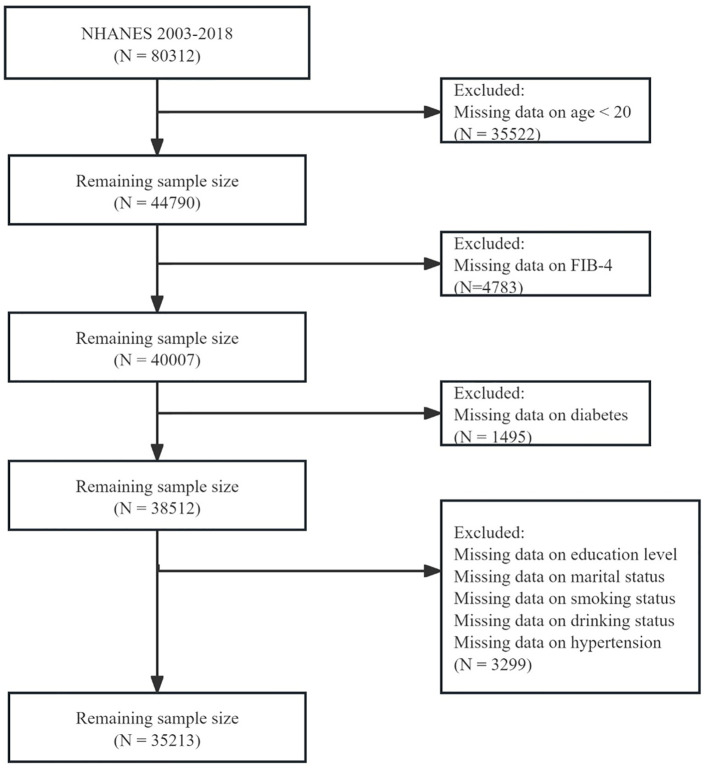
Flowchart of participant selection in NHANES 2003-2018. The figure summarizes exclusion steps based on age eligibility, FIB-4 availability, diabetes outcome availability, survey design variables, and covariates used in the analytic models.

**Figure 2:**
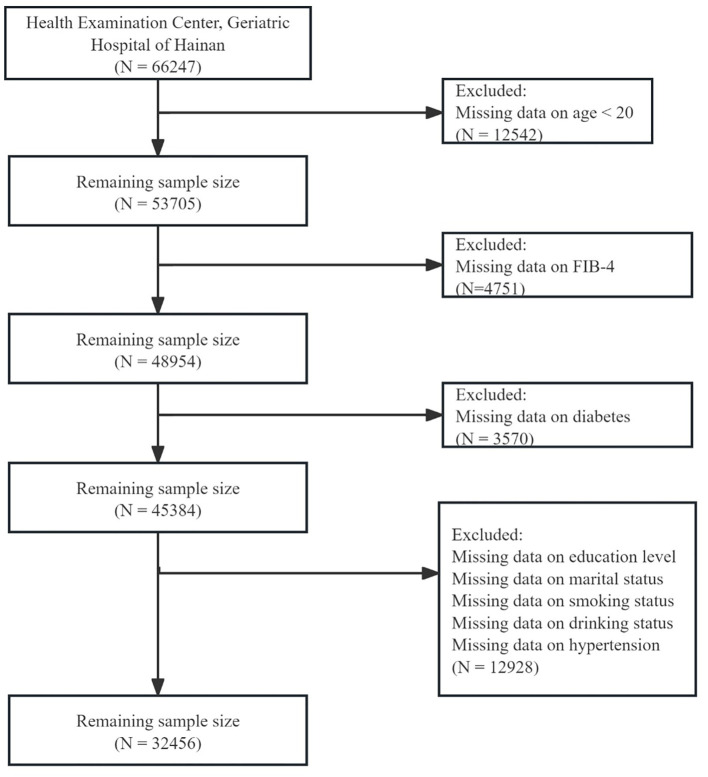
Flowchart of participant selection in the hospital-based health examination population, 2022-2025. The figure summarizes exclusions based on age eligibility, FIB-4 availability, diabetes outcome availability, and covariates used in the analytic models.

The second population included adults who underwent consecutive routine health examinations at the Health Examination Center of the Geriatric Hospital of Hainan between 2022 and 2025. Data were obtained from electronic health examination records. After the corresponding eligibility criteria were applied, 32,456 adults were included in the hospital-based analysis. This population was used to examine whether the association observed in NHANES showed a broadly similar pattern in an independent hospital-based health examination setting rather than as a population-representative external validation cohort. The participant selection processes are shown in **Figures 1** and **2**. The hospital-based component was approved by the Ethics Committee of the Geriatric Hospital of Hainan (approval No. EAN-2025-047).

### 2.2 Exposure assessment

FIB-4 was calculated using the following formula: (11)


$$FIB-4=\frac{Age\left(years\right)\times AST\left(U/L\right)}{PLT\left(10^{9}/L\right)\times \sqrt{ALT\left(U/L\right)}}$$

In NHANES, AST and ALT were measured using an enzymatic rate method with a Beckman Coulter MAXM chemistry analyzer, and platelet count was measured using electrical impedance with a Beckman Coulter MAXM hematology analyzer. In the hospital-based health examination population, AST and ALT were measured using an enzymatic rate method with a Hitachi automated biochemical analyzer, and platelet count was measured using electrical impedance with a Mindray automated hematology analyzer.

### 2.3 Outcome assessment

Prevalent diabetes was the outcome variable. In NHANES, prevalent diabetes was defined as self-reported physician-diagnosed diabetes, current use of insulin or oral glucose-lowering medications, HbA1c ≥6.5%, or fasting plasma glucose ≥126 mg/dL when available (12). In the hospital-based health examination population, prevalent diabetes was ascertained from documented clinical diagnoses, medication records, or available laboratory criteria recorded in electronic health examination records, including HbA1c ≥6.5% or fasting plasma glucose ≥126 mg/dL when available.

### 2.4 Covariates

Covariates were selected based on prior literature, clinical relevance, and epidemiologic considerations. Demographic variables included age, sex, race/ethnicity, education level, and marital status; race/ethnicity was available only in NHANES. Socioeconomic status was assessed using the family income-to-poverty ratio when available (13, 14). Lifestyle variables included smoking status and alcohol consumption status. Metabolic and clinical variables included body mass index (BMI), hypertension, and total cholesterol. Selected laboratory variables included urinary creatinine, white blood cell count, hemoglobin, and serum creatinine when available. Because age is included in the FIB-4 formula and is strongly associated with prevalent diabetes, we retained age adjustment in multivariable models and performed additional sensitivity analyses using continuous age and narrower age strata. The NHANES analysis incorporated official sampling weights, strata, and primary sampling units to account for the complex survey design.

### 2.5 Statistical analysis

Survey-weighted multivariable logistic regression was used for NHANES to evaluate the association between FIB-4 and prevalent diabetes. The 2-year mobile examination center weights were recalculated by dividing WTMEC2YR by 8, and strata and primary sampling units were incorporated to account for the complex sampling design. Conventional multivariable logistic regression was used for the hospital-based population under a comparable covariate framework. Three sequential models were fitted. In NHANES, Model 1 was unadjusted; Model 2 was adjusted for age group (<50 and ≥50 years) and sex; and Model 3 was further adjusted for race/ethnicity, education level, marital status, income-to-poverty ratio, alcohol consumption, BMI, hypertension, smoking status, total cholesterol, urinary creatinine, white blood cell count, hemoglobin, serum creatinine, and age group (<50 and ≥50 years). In the hospital-based population, Model 2 was adjusted for continuous age and sex, and Model 3 was further adjusted for race/ethnicity, education level, marital status, smoking status, alcohol consumption, BMI, hypertension, total cholesterol, serum creatinine, white blood cell count, hemoglobin, and continuous age. FIB-4 was analyzed both continuously and by tertiles, with tertile 1 used as the reference group. *P* for trend was calculated by entering the tertile variable into the model as an ordinal term.

Nonlinear associations were examined using restricted cubic spline (RCS) models with four knots fitted using the rms package. The knots were placed at the 5th, 35th, 65th, and 95th percentiles of the FIB-4 distribution, following a commonly used Harrell knot-placement strategy for four-knot restricted cubic splines (45). The RCS models were fitted under the fully adjusted covariate framework. Two-piecewise logistic regression was used only as an exploratory statistical description of the fitted curve. The inflection point was identified using an Akaike information criterion-based grid-search procedure, in which 200 candidate FIB-4 values between the 5th and 95th percentiles of the FIB-4 distribution were evaluated. These inflection points were not interpreted as clinical thresholds for screening, diagnosis, decision-making, or intervention.

Additional analyses were performed to address age-related structural dependence, residual confounding, and clinical utility (15, 16). We calculated collinearity diagnostics in models containing FIB-4 and continuous age and additionally fitted models containing both linear and quadratic age terms. Because the primary NHANES models used age-group adjustment, we repeated the fully adjusted NHANES models after replacing age group with continuous age; the hospital-based Model 3 already used continuous age and was therefore checked as a model-specification and collinearity analysis rather than as a separate continuous-age sensitivity model. We also conducted analyses within narrower age bands (18-39, 40-49, 50-59, 60-69, and ≥70 years) and examined age-free liver-related indices, including the AST-to-platelet ratio index (APRI) and AST/ALT ratio. To assess incremental model performance, we fitted age-only benchmark models and compared models containing traditional diabetes predictors with and without FIB-4 using AUC and Brier score; calibration plots, integrated discrimination improvement, continuous net reclassification improvement, and decision-curve analyses were treated as exploratory summaries. Additional NHANES sensitivity analyses excluded participants with self-reported liver conditions and those with extreme AST, ALT, or platelet values. Variable-specific missingness before construction of the final analytic datasets was summarized (**Supplementary Table S7**).

## 3 Results

### 3.1 Baseline characteristics

A total of 35,213 adults from NHANES 2003–2018 were included, representing approximately 187 million noninstitutionalized US adults after survey weighting. The weighted prevalence of diabetes was 12.61%. Across increasing FIB-4 tertiles, the prevalence of diabetes increased from 5.93% in tertile 1 to 12.46% in tertile 2 and 21.35% in tertile 3. Participants in higher FIB-4 tertiles were older and had a higher prevalence of hypertension, underscoring the need to interpret FIB-4 associations in the context of age and cardiometabolic comorbidity. Weighted baseline characteristics are shown in **Table 1**.

**Table 1:** Survey-weighted baseline characteristics of adults by FIB-4 tertiles in NHANES 2003–2018.

Characteristic	N^1^	Overall N = 187,086,349^2^	T1 N = 68,101,785^2^	T2 N = 65,963,418^2^	T3 N = 53,021,147^2^	*P* value^3^
Age (years)	35,213	46.00 (33.00, 60.00)	30.00 (25.00, 38.00)	48.00 (40.00, 56.00)	65.00 (56.00, 74.00)	<0.001
Gender (%)	35,213					<0.001
Male		17,370 (48.95%)	5,150 (46.23%)	5,711 (49.17%)	6,509 (52.16%)	
Female		17,843 (51.05%)	6,588 (53.77%)	6,026 (50.83%)	5,229 (47.84%)	
Race (%)	35,213					<0.001
Mexican American		5,825 (8.43%)	2,352 (12.05%)	1,969 (7.86%)	1,504 (4.49%)	
Other Hispanic		3,056 (5.17%)	1,049 (6.60%)	1,125 (5.24%)	882 (3.25%)	
Non-Hispanic White		15,790 (69.01%)	4,688 (61.75%)	5,012 (69.56%)	6,090 (77.64%)	
Non-Hispanic Black		7,084 (10.39%)	2,336 (11.46%)	2,430 (10.29%)	2,318 (9.15%)	
Other Race		3,458 (7.00%)	1,313 (8.15%)	1,201 (7.04%)	944 (5.47%)	
Education level (%)	35,213					<0.001
Less Than 9th Grade		3,719 (5.26%)	721 (3.81%)	1,226 (5.13%)	1,772 (7.28%)	
9-11th Grade		4,941 (10.36%)	1,735 (11.14%)	1,597 (9.66%)	1,609 (10.24%)	
High School Grad/GED or Equivalent		8,160 (23.67%)	2,747 (23.88%)	2,638 (22.89%)	2,775 (24.39%)	
Some College or AA degree		10,382 (31.60%)	4,033 (35.32%)	3,334 (30.27%)	3,015 (28.49%)	
College Graduate or above		8,011 (29.10%)	2,502 (25.84%)	2,942 (32.05%)	2,567 (29.60%)	
Marital Status (%)	35,213					<0.001
married		18,454 (56.12%)	4,889 (43.90%)	6,872 (63.36%)	6,693 (62.82%)	
Widowed		2,817 (5.66%)	98 (0.75%)	577 (3.77%)	2,142 (14.30%)	
Divorced		3,746 (10.19%)	715 (6.33%)	1,560 (12.49%)	1,471 (12.28%)	
Separated		1,106 (2.27%)	361 (2.48%)	453 (2.67%)	292 (1.52%)	
Never married		6,272 (17.72%)	4,080 (33.62%)	1,441 (11.01%)	751 (5.65%)	
Living with partner		2,818 (8.04%)	1,595 (12.92%)	834 (6.70%)	389 (3.44%)	
Drinking (%)	35,213					<0.001
Yes		9,394 (21.61%)	2,827 (19.74%)	3,077 (20.66%)	3,490 (25.20%)	
No		25,819 (78.39%)	8,911 (80.26%)	8,660 (79.34%)	8,248 (74.80%)	
Hypertension (%)	35,213					<0.001
No		17,528 (53.98%)	8,436 (71.85%)	5,708 (51.99%)	3,384 (33.52%)	
Yes		17,685 (46.02%)	3,302 (28.15%)	6,029 (48.01%)	8,354 (66.48%)	
Smoking(%)	35,213					<0.001
No		19,249 (54.41%)	7,085 (58.01%)	6,364 (53.70%)	5,800 (50.65%)	
Smoking now		7,273 (20.63%)	2,947 (25.65%)	2,619 (21.34%)	1,707 (13.32%)	
Once a smoker		8,691 (24.96%)	1,706 (16.33%)	2,754 (24.96%)	4,231 (36.04%)	
BMI (kg/m^2^)	35,213	27.80 (24.15, 32.30)	27.97 (23.90, 33.00)	27.90 (24.37, 32.21)	27.50 (24.20, 31.60)	<0.001
Total Cholesterol (mg/dL)	35,213	192.00 (166.00, 220.00)	186.00 (161.00, 213.00)	198.00 (172.00, 226.00)	193.00 (165.00, 222.00)	<0.001
Hemoglobin (g/dL)	35,213	14.30 (13.40, 15.30)	14.30 (13.30, 15.40)	14.40 (13.50, 15.40)	14.20 (13.30, 15.20)	<0.001
Creatinine (umol/L)	35,213	76.02 (63.65, 88.40)	70.72 (61.88, 83.10)	76.02 (64.53, 88.40)	80.44 (68.95, 96.36)	<0.001
Diabetes (%)	35,213					<0.001
No		29,255 (87.39%)	10,893 (94.07%)	9,701 (87.54%)	8,661 (78.65%)	
Yes		5,958 (12.61%)	845 (5.93%)	2,036 (12.46%)	3,077 (21.35%)	
FIB-4	35,213	0.89 (0.60, 1.32)	0.53 (0.43, 0.63)	0.95 (0.83, 1.08)	1.66 (1.42, 2.05)	<0.001

^1^ N not Missing (unweighted).

^2^ Median (Q1, Q3); n (unweighted) (%).

^3^ Design-based Kruskal–Wallis test; Rao–Scott adjusted χ² test: Rao & Scott adjustment.

The hospital-based health examination population included 32,456 adults from the Geriatric Hospital of Hainan between 2022 and 2025. The prevalence of diabetes increased across FIB-4 tertiles, from 5.58% in tertile 1 to 11.78% in tertile 2 and 20.62% in tertile 3. Similar to the NHANES population, participants in higher FIB-4 tertiles were older and had a higher prevalence of hypertension. Baseline characteristics are shown in **Table 2**.

**Table 2:** Baseline characteristics of adults by FIB-4 tertiles in the hospital-based health examination population, 2022–2025.

Characteristic	N^1^	Overall N = 32,456^2^	T1 N = 10,819^2^	T2 N = 10,818^2^	T3 N = 10,819^2^	*P* value^3^
Age (years)	32,456	46.00 (35.00, 59.00)	33.00 (28.00, 39.00)	46.00 (39.00, 54.00)	63.00 (55.00, 71.00)	<0.001
Gender (%)	32,456					<0.001
Male		15,800 (48.68%)	4,905 (45.34%)	5,344 (49.40%)	5,551 (51.31%)	
Female		16,656 (51.32%)	5,914 (54.66%)	5,474 (50.60%)	5,268 (48.69%)	
Education level (%)	32,456					<0.001
Elementary School		1,684 (5.19%)	417 (3.85%)	536 (4.95%)	731 (6.76%)	
Middle School		3,407 (10.50%)	1,219 (11.27%)	1,095 (10.12%)	1,093 (10.10%)	
High School		7,676 (23.65%)	2,482 (22.94%)	2,519 (23.29%)	2,675 (24.73%)	
University		19,689 (60.66%)	6,701 (61.94%)	6,668 (61.64%)	6,320 (58.42%)	
Marital Status (%)	32,456					<0.001
married		18,363 (56.58%)	4,863 (44.95%)	6,610 (61.10%)	6,890 (63.68%)	
Widowed		1,786 (5.50%)	88 (0.81%)	426 (3.94%)	1,272 (11.76%)	
Divorced		3,358 (10.35%)	746 (6.90%)	1,265 (11.69%)	1,347 (12.45%)	
Separated		734 (2.26%)	273 (2.52%)	268 (2.48%)	193 (1.78%)	
Never married		5,640 (17.38%)	3,493 (32.29%)	1,472 (13.61%)	675 (6.24%)	
Living with partner		2,575 (7.93%)	1,356 (12.53%)	777 (7.18%)	442 (4.09%)	
Drinking (%)	32,456					<0.001
Yes		25,500 (78.57%)	8,713 (80.53%)	8,614 (79.63%)	8,173 (75.54%)	
No		6,956 (21.43%)	2,106 (19.47%)	2,204 (20.37%)	2,646 (24.46%)	
Smoking(%)	32,456					<0.001
No		17,651 (54.38%)	6,273 (57.98%)	5,778 (53.41%)	5,600 (51.76%)	
Smoking now		8,161 (25.14%)	1,846 (17.06%)	2,676 (24.74%)	3,639 (33.64%)	
Once a smoker		6,644 (20.47%)	2,700 (24.96%)	2,364 (21.85%)	1,580 (14.60%)	
Hypertension (%)	32,456					<0.001
No		17,504 (53.93%)	7,694 (71.12%)	5,806 (53.67%)	4,004 (37.01%)	
Yes		14,952 (46.07%)	3,125 (28.88%)	5,012 (46.33%)	6,815 (62.99%)	
BMI (kg/m2)	32,456	28.06 (24.24, 32.13)	28.27 (24.14, 32.74)	28.09 (24.29, 32.07)	27.85 (24.28, 31.60)	<0.001
Total Cholesterol (mg/dL)	32,456	192.28(166.02, 218.91)	187.26 (161.39, 211.97)	196.14 (155.98, 222.78)	193.82 (166.41, 222.01)	<0.001
Creatinine (umol/L)	32,456	74.90 (64.00, 88.10)	70.80 (61.40, 82.30)	75.10 (64.10, 87.90)	79.50 (67.60, 93.70)	<0.001
Hemoglobin (g/dL)	32,456	14.34 (13.37, 15.29)	14.39 (13.38, 15.37)	14.41 (13.47, 15.34)	14.23 (13.28, 15.17)	<0.001
Diabetes (%)	32,456					<0.001
No		28,347 (87.34%)	10,215 (94.42%)	9,544 (88.22%)	8,588 (79.38%)	
Yes		4,109 (12.66%)	604 (5.58%)	1,274 (11.78%)	2,231 (20.62%)	
FIB-4	32,456	0.85 (0.61, 1.27)	0.52 (0.43, 0.61)	0.85 (0.75, 0.97)	1.51 (1.27, 1.84)	<0.001

^1^ N Non-missing.

^2^ Median (Q1, Q3); n (%).

^3^ Kruskal-Wallis rank sum test; Pearson’s Chi-squared test.

### 3.2 Association between the FIB-4 index and prevalent diabetes

In NHANES, each 1-unit increase in FIB-4 was associated with higher odds of prevalent diabetes in the unadjusted model (OR, 1.64; 95% CI, 1.49-1.81; *P* < 0.001). The corresponding ORs were 1.12 (95% CI, 1.05-1.20; *P* < 0.001) in Model 2 and 1.13 (95% CI, 1.06-1.22; *P* < 0.001) in Model 3. In the fully adjusted tertile-based analysis using age-group adjustment (<50 and ≥50 years), compared with tertile 1, the ORs were 1.38 (95% CI, 1.19-1.59) for tertile 2 and 1.66 (95% CI, 1.40-1.96) for tertile 3 (*P* for trend< 0.001). These results are shown in **Table 3**; continuous-age sensitivity results are described below.

**Table 3:** Association between FIB-4 and prevalent diabetes in NHANES 2003–2018.

	Model 1 OR (95% CI) *P* value	Model 2 OR (95% CI) *P* value	Model 3 OR (95% CI) *P* value
FIB-4 (continuous)	1.642 (1.490, 1.810)<0.001	1.122 (1.048, 1.200)<0.001	1.134 (1.057, 1.217)<0.001
T1	[Reference]	[Reference]	[Reference]
T2	2.257 (2.041, 2.497)<0.001	1.125 (0.982, 1.289) 0.089	1.377 (1.190, 1.593)<0.001
T3	4.305 (3.900, 4.753)<0.001	1.311 (1.130, 1.520)<0.001	1.660 (1.404, 1.962)<0.001
*P* for trend	<0.001	<0.001	<0.001

Model 1 was unadjusted. Model 2 was adjusted for age group (<50 and ≥50 years) and sex. Model 3 was adjusted for age group (<50 and ≥50 years), sex, race/ethnicity, education level, marital status, income-to-poverty ratio, alcohol consumption, BMI, hypertension, smoking status, total cholesterol, urinary creatinine, white blood cell count, hemoglobin, and serum creatinine. NHANES sampling weights, strata, and primary sampling units were incorporated.

In the hospital-based health examination population, each 1-unit increase in FIB-4 was associated with higher odds of prevalent diabetes in the unadjusted model (OR, 2.56; 95% CI, 2.42-2.71; *P* < 0.001). The corresponding ORs were 1.42 (95% CI, 1.31-1.54; *P* < 0.001) in Model 2 and 1.36 (95% CI, 1.25-1.48; *P* < 0.001) in Model 3, which used continuous age. In the fully adjusted tertile-based analysis, compared with tertile 1, the ORs were 1.51 (95% CI, 1.35-1.70) for tertile 2 and 1.76 (95% CI, 1.53-2.03) for tertile 3 (*P* for trend< 0.001). These results are shown in **Table 4**.

**Table 4:** Association between FIB-4 and prevalent diabetes in the hospital-based health examination population, 2022–2025.

	Model 1 OR (95% CI) *P* value	Model 2 OR (95% CI) *P* value	Model 3 OR (95% CI) *P* value
FIB-4 (continuous)	2.562 (2.422, 2.710)<0.001	1.417 (1.305, 1.537)<0.001	1.358 (1.245, 1.480)<0.001
T1	[Reference]	[Reference]	[Reference]
T2	2.258 (2.044, 2.498)<0.001	1.514 (1.356, 1.691)<0.001	1.511 (1.345, 1.698)<0.001
T3	4.393 (4.001, 4.832)<0.001	1.876 (1.641, 2.144)<0.001	1.761 (1.528, 2.030)<0.001
*P* for trend	<0.001	<0.001	<0.001

Model 1 was unadjusted. Model 2 was adjusted for continuous age and sex. Model 3 was adjusted for continuous age, sex, race/ethnicity, education level, marital status, smoking status, alcohol consumption, BMI, hypertension, total cholesterol, serum creatinine, white blood cell count, and hemoglobin.

### 3.3 Nonlinear association and two-piecewise regression analysis

**Figure 3:**
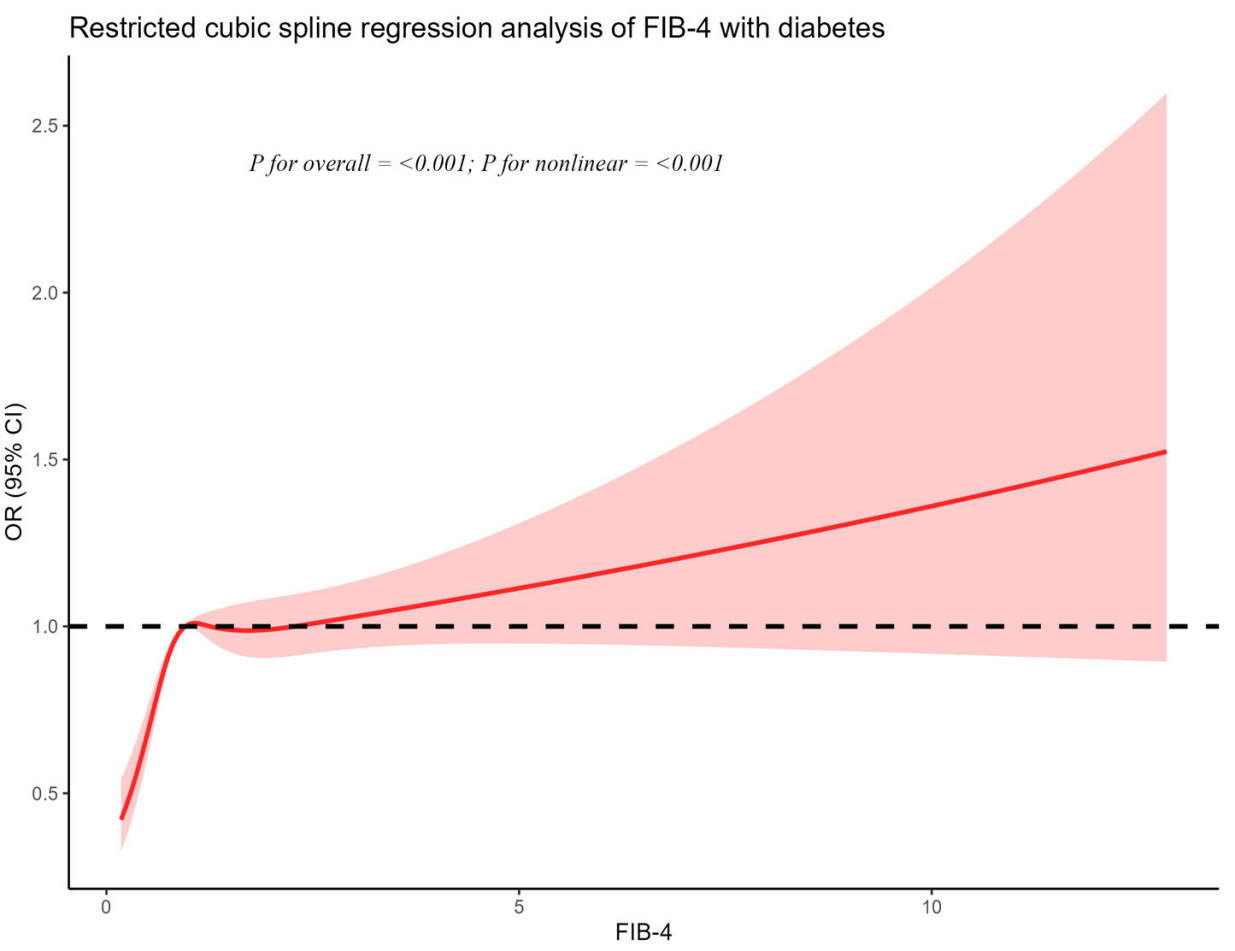
Restricted cubic spline curve for the association between FIB-4 and prevalent diabetes in NHANES 2003-2018. Odds ratios were estimated using survey-weighted logistic regression under the fully adjusted covariate framework. The curve is descriptive and should not be interpreted as evidence of a clinical threshold.

**Figure 4:**
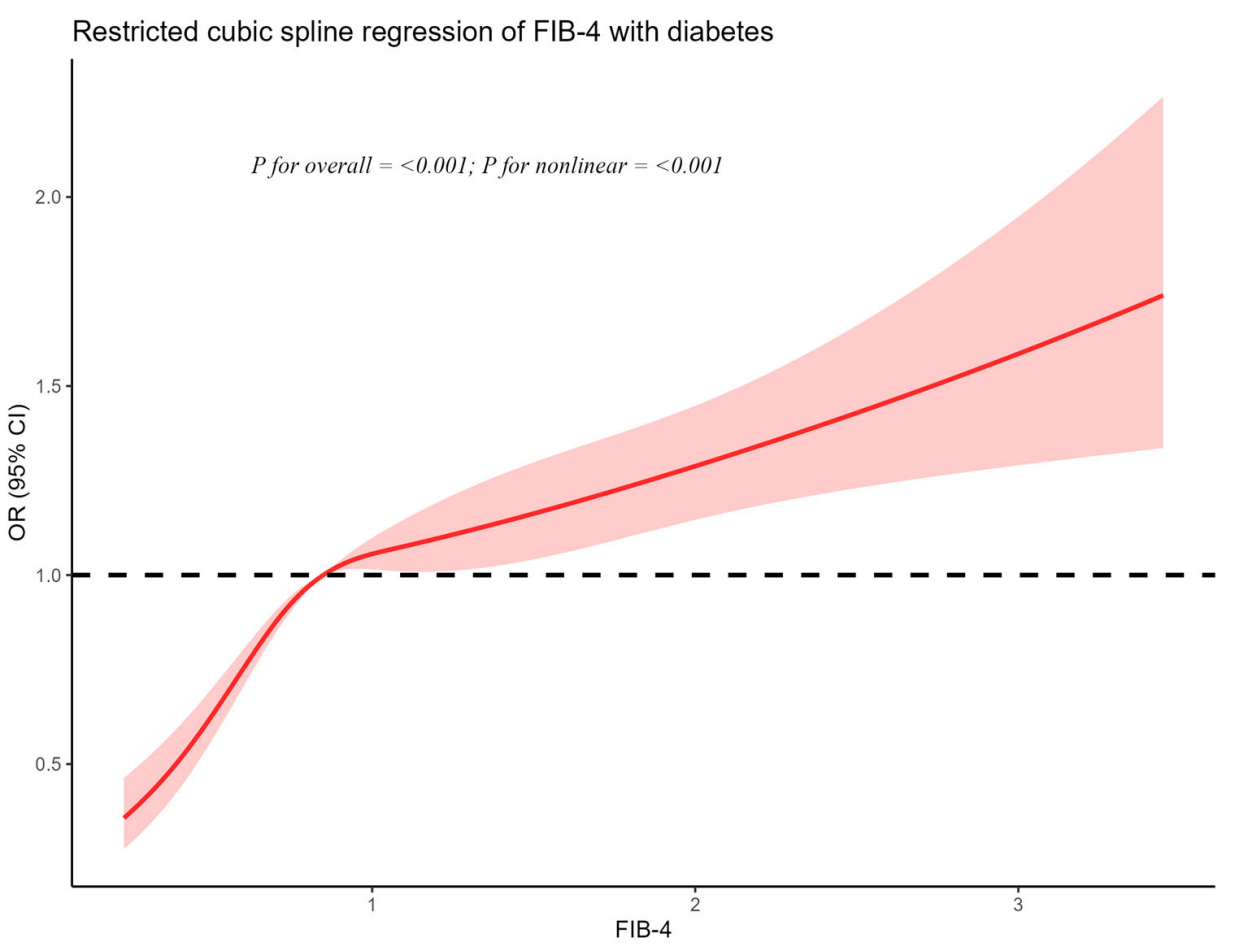
Restricted cubic spline curve for the association between FIB-4 and prevalent diabetes in the hospital-based health examination population. Odds ratios were estimated using multivariable logistic regression under the fully adjusted covariate framework. The curve is descriptive and should not be interpreted as evidence of a clinical threshold.

Restricted cubic spline analyses were performed to describe the nonlinear association between FIB-4 and prevalent diabetes. In both NHANES and the hospital-based health examination population, the tests for nonlinearity were statistically significant (*P* for nonlinearity< 0.001). These curves should be interpreted as descriptive statistical patterns rather than evidence of threshold biology. The restricted cubic spline results are shown in **Figures 3** and **4**.

In exploratory two-piecewise logistic regression models, the model-derived statistical inflection point was 0.737 in NHANES and 0.745 in the hospital-based health examination population. These values were below commonly used clinical FIB-4 cutoffs for advanced fibrosis and therefore have no direct clinical threshold implication. The two-piecewise regression results were moved to **Supplementary Tables S8** and **S9**.

### 3.4 Subgroup analyses and interaction tests

Subgroup analyses were performed according to sex, age group, hypertension status, obesity status, smoking status, and alcohol consumption status. In NHANES, the associations between FIB-4 tertiles and prevalent diabetes were evaluated within each prespecified subgroup. Interaction tests were statistically significant for sex, age group, hypertension status, and obesity status, whereas no statistically significant interaction was observed for smoking status or alcohol consumption status. Because these analyses were exploratory, the subgroup results for NHANES were moved to **Supplementary Figure S3**.

In the hospital-based health examination population, subgroup analyses were performed using the same prespecified stratification variables, except for variables unavailable or defined differently in the dataset. A statistically significant interaction was observed for obesity status, whereas no statistically significant interaction was observed for sex, age group, hypertension status, smoking status, or alcohol consumption status. The exploratory subgroup results for the hospital-based health examination population were moved to **Supplementary Figure S4**.

### 3.5 Sensitivity and incremental model-performance analyses

Collinearity diagnostics did not indicate problematic statistical multicollinearity between FIB-4 and continuous age. In NHANES, the VIFs were 1.28 for FIB-4 and 1.52 for age. In the hospital-based population, the corresponding adjusted GVIF metrics were 1.41 and 1.44, respectively. After age group was replaced with continuous age in NHANES, the association was substantially attenuated: the OR per 1-unit increase in FIB-4 was 1.03 (95% CI, 0.95-1.12; *P* = 0.518). The categorical estimates were also attenuated and did not show a clear monotonic gradient. In contrast, the hospital-based Model 3 already used continuous age and remained positive for FIB-4 analyzed continuously (OR, 1.36; 95% CI, 1.25-1.48; *P* < 0.001), T2 versus T1 (OR, 1.51; 95% CI, 1.35-1.70; *P* < 0.001), and T3 versus T1 (OR, 1.76; 95% CI, 1.53-2.03; *P* < 0.001). Narrow age-band analyses were heterogeneous in both datasets, with positive associations observed in only some age strata. In models that included both linear and quadratic age terms, FIB-4 was not statistically significant in NHANES (OR, 1.05; 95% CI, 0.98-1.13; *P* = 0.158), whereas the hospital-based association persisted but was attenuated (OR, 1.33; 95% CI, 1.22-1.45; *P* < 0.001; **Supplementary Table S11**). These findings indicate that the age-adjusted association was not consistent across the two datasets (**Supplementary Tables S1**, **S4**, **S10**, **S11**).

Exploratory model-performance analyses showed little incremental value of FIB-4. Age-only benchmark models yielded AUCs of 0.730 in NHANES and 0.682 in the hospital-based population. In NHANES, the AUC was 0.822 for the model containing traditional predictors and remained 0.822 after FIB-4 was added; the Brier score was essentially unchanged (0.0914 vs 0.0914). In the hospital-based population, the AUC increased only minimally from 0.756 to 0.758, with no material change in the Brier score (0.0992 vs 0.0990). IDI and continuous NRI estimates were negligible or inconsistent across datasets, and calibration plots and decision-curve analyses did not support the use of FIB-4 as an independent diabetes prediction tool (**Supplementary Tables S2**, **S3**; **Supplementary Figures S1**, **S2**).

Age-free alternative indices showed modest associations with prevalent diabetes, but their direction and magnitude were not fully consistent across datasets (**Supplementary Table S5**). In NHANES, excluding participants with self-reported liver conditions or extreme AST, ALT, or platelet values attenuated or reversed the FIB-4 association (**Supplementary Table S6**), indicating that the association was not robust to liver-related exclusions.

## 4 Discussion

### 4.1 Main findings

This study evaluated the association between FIB-4 and prevalent diabetes in a nationally representative US sample and an independent Chinese hospital-based health examination population. NHANES models using age groups suggested a positive association, but the association was attenuated to the null after age was modeled continuously. In contrast, the hospital-based association remained positive in models that already included continuous age. Thus, the observed relationship cannot be uniformly attributed to age alone, but neither can it be regarded as a consistently age-independent association. Moreover, FIB-4 provided little or no incremental model performance beyond traditional diabetes predictors. Together, these findings support a cautious, population- and setting-dependent interpretation rather than a clinical screening or prediction role for FIB-4.

### 4.2 Biological and epidemiologic interpretation

FIB-4 should be viewed as a composite liver-related index rather than as a diabetes-specific biomarker (17–27). AST, ALT, platelet count, and age may reflect hepatocellular injury, platelet-related changes, aging, and metabolic comorbidity, but the present cross-sectional data cannot determine whether these processes precede diabetes, result from diabetes, or simply co-occur with shared cardiometabolic risk factors (28–34).

The age component of FIB-4 is central to interpretation because age directly increases the index and is also strongly associated with diabetes prevalence. The low VIF and GVIF values indicate that the simultaneous inclusion of FIB-4 and age did not cause major numerical instability, but statistical collinearity is distinct from structural dependence. These diagnostics therefore do not eliminate residual age-related confounding or the interpretive circularity created by embedding age in the exposure definition. The divergent continuous-age results across datasets may reflect differences in sampling design, age distribution, diabetes ascertainment, laboratory platforms, covariate availability, liver disease prevalence, medication use, and selection mechanisms. NHANES is nationally representative and incorporates complex survey weighting, whereas the Chinese dataset was derived from a hospital health examination population and may be affected by healthy-worker effects, socioeconomic selection, referral patterns, and metabolic enrichment. These differences argue against interpreting the hospital-based estimate as definitive evidence of an age-independent biological association.

The nonlinear and two-piecewise findings should also be interpreted conservatively. The model-derived inflection points were below established FIB-4 clinical cutoffs for advanced fibrosis, and their location may depend on the age distribution, covariate structure, and FIB-4 distribution of each dataset. They should therefore be regarded as exploratory statistical descriptions rather than clinically actionable cutoffs.

### 4.3 Relationship with previous studies

Previous studies have described close interrelations among diabetes, insulin resistance, metabolic dysfunction-associated steatotic liver disease, and liver fibrosis progression (35–38). Current guidelines recommend FIB-4 as an accessible first-line blood-based tool for liver fibrosis risk stratification in individuals with metabolic abnormalities, but this liver-fibrosis use case is distinct from diabetes screening or prediction.

Most existing evidence has evaluated FIB-4 in individuals with established diabetes, obesity, or metabolic dysfunction-associated steatotic liver disease, primarily in relation to advanced fibrosis, cardiovascular outcomes, liver-related events, or mortality (39, 40). The present study differs by examining FIB-4 as an exposure marker associated with prevalent diabetes in broader adult populations and by explicitly evaluating age-related circularity and incremental model performance.

Many previous analyses have classified FIB-4 using conventional liver fibrosis cutoffs (41). Although this approach is clinically appropriate for liver disease referral pathways, it is less informative for characterizing the continuous relationship between FIB-4 and prevalent diabetes. The present analyses indicate that continuous modeling can describe epidemiologic patterns, but these patterns should not be translated into diabetes risk thresholds without prospective validation.

### 4.4 Clinical and public health implications

The FIB-4 index is calculated from routinely available laboratory variables and is easy to obtain in health examination and primary care settings. Nevertheless, accessibility alone does not establish clinical utility. In the present exploratory performance analyses, adding FIB-4 to traditional diabetes predictors did not improve discrimination in NHANES and produced only a very small AUC increase in the hospital-based population.

Accordingly, FIB-4 may alert clinicians to coexisting liver-related abnormalities in people with metabolic risk factors, but it should not be used to screen for or predict diabetes independently of established diagnostic tests. Clinical assessment should continue to rely on fasting plasma glucose, glycated hemoglobin, oral glucose tolerance testing when indicated, and comprehensive cardiometabolic evaluation.

Future studies should use prospective designs, adjudicated incident diabetes outcomes, more complete liver disease phenotyping, and formal prediction-model development and validation frameworks before considering any diabetes-related risk stratification role for FIB-4 (42–44).

### 4.5 Strengths and limitations

This study has several strengths. It included a large nationally representative US sample and an independent Chinese hospital-based health examination population, allowing comparison of association patterns across distinct data sources. FIB-4 was evaluated as both a continuous and categorical variable, with nonlinear associations examined using RCS models. The NHANES analysis incorporated sampling weights, strata, and primary sampling units. Additional analyses quantified collinearity, age-related sensitivity, age-free alternative indices, calibration, reclassification, and decision-curve metrics.

Several limitations should be acknowledged. First, the cross-sectional design precludes assessment of temporality; reverse causality cannot be excluded because diabetes itself may alter AST, ALT, platelet count, inflammation, and hepatic metabolism. Second, age is embedded in FIB-4, and residual confounding may persist if the age-diabetes relationship is nonlinear or differs across populations, although additional models including an age-squared term showed the same qualitative cross-dataset pattern. The different estimates obtained in NHANES and the hospital-based population indicate that the association is not fully transportable across settings. Third, liver-related confounders were incompletely captured. NHANES allowed exclusion of self-reported liver disease and extreme AST/ALT/platelet values, and the association was not robust to liver-related exclusions; however, detailed viral hepatitis status, steatotic liver disease severity, hepatotoxic medication exposure, cirrhosis, and chronic inflammatory disorders were not fully available across both datasets. Fourth, the hospital-based health examination population was not population based and may be affected by referral bias, healthy-worker bias, socioeconomic selection, and metabolic enrichment; it should therefore be considered a reproducibility dataset rather than true external population validation. Fifth, complete-case restriction may have introduced selection bias; variable-specific missingness was summarized before construction of the final analytic datasets, but these summaries do not establish that missingness was completely at random or missing at random. Sixth, the model-derived inflection points have no direct clinical threshold implication. Finally, the exploratory model-performance analyses showed limited incremental value of FIB-4 beyond traditional diabetes predictors, particularly in NHANES.

## 5 Conclusions

In this cross-sectional analysis, the association between FIB-4 and prevalent diabetes differed according to age parameterization and study population. The association was attenuated and no longer statistically significant after continuous-age adjustment in NHANES, whereas it persisted in the hospital-based population where the primary model already used continuous age. Nevertheless, FIB-4 added little or no incremental model performance beyond traditional diabetes predictors. These findings are associative and setting-dependent and do not support the use of FIB-4 as an independent screening, diagnostic, or predictive tool for diabetes.

## Data Availability

Publicly available datasets were analyzed in this study. This data can be found here: The NHANES datasets analyzed in this study are publicly available from the National Center for Health Statistics: https://www.cdc.gov/nchs/nhanes/about/index.html. The HECGHH data are not publicly available because of institutional and privacy restrictions, but may be available from the corresponding author upon reasonable request and with appropriate institutional approval.
