# Supplementary material for: Association of the fibrosis-4 index with prevalent diabetes among adults: a cross-sectional analysis of NHANES 2003–2018 and a Chinese hospital-based health examination population
Source: Front Endocrinol (Lausanne). 2026 Jul 30;17:1881759. doi: 10.3389/fendo.2026.1881759 (PMC13467833; doi:10.3389/fendo.2026.1881759)
Supplement: Supplementary file 1 [file DataSheet1.docx]

**Supplementary Material**

**Supplementary Table S1.** NHANES continuous-age sensitivity models.

| **Model** | **Term** | **OR** | **95% CI low** | **95% CI high** | **P value** |
| --- | --- | --- | --- | --- | --- |
| Minimally adjusted continuous FIB-4 model | FIB-4, per 1-unit increase | 1.000 | 0.917 | 1.091 | 0.996 |
| Fully adjusted continuous FIB-4 model | FIB-4, per 1-unit increase | 1.027 | 0.946 | 1.116 | 0.518 |
| Minimally adjusted FIB-4 tertile model | T2 vs T1 | 1.113 | 0.973 | 1.274 | 0.117 |
| Minimally adjusted FIB-4 tertile model | T3 vs T1 | 1.002 | 0.843 | 1.190 | 0.983 |
| Fully adjusted FIB-4 tertile model | T2 vs T1 | 1.235 | 1.074 | 1.419 | 0.003 |
| Fully adjusted FIB-4 tertile model | T3 vs T1 | 1.185 | 0.992 | 1.415 | 0.061 |

S1 note: Minimally adjusted models included continuous age and sex; fully adjusted models additionally included race/ethnicity, education level, marital status, income-to-poverty ratio, alcohol consumption, BMI, hypertension, smoking status, total cholesterol, urinary creatinine, white blood cell count, hemoglobin, and serum creatinine. NHANES sampling weights, strata, and primary sampling units were incorporated. T1 was the reference group.

**Supplementary Table S2. Model performance for age-only benchmark and traditional predictor models with and without FIB-4.**

| **Dataset** | **Model** | **N** | **AUC** | **Brier score** |
| --- | --- | --- | --- | --- |
| NHANES | Age alone | 35213 | 0.730 | 0.1034 |
| NHANES | Traditional predictors | 35213 | 0.822 | 0.0914 |
| NHANES | Traditional predictors + FIB-4 | 35213 | 0.822 | 0.0914 |
| Hospital health-examination cohort | Age alone | 32456 | 0.682 | 0.1061 |
| Hospital health-examination cohort | Traditional predictors | 32456 | 0.756 | 0.0992 |
| Hospital health-examination cohort | Traditional predictors + FIB-4 | 32456 | 0.758 | 0.0990 |

**Supplementary Table S3. Incremental discrimination and reclassification.**

| Dataset | IDI | Continuous NRI | Event component | Non-event component |
| --- | --- | --- | --- | --- |
| NHANES | -0.000 | -0.183 | -0.249 | 0.065 |
| Hospital health-examination cohort | 0.002 | 0.060 | -0.086 | 0.145 |

**Supplementary Table S4. Narrow age-band sensitivity analyses.**

| Dataset | Age band | Term | OR | 95% CI low | 95% CI high | P value | N | Events |
| --- | --- | --- | --- | --- | --- | --- | --- | --- |
| NHANES | 18-39 | FIB | 1.084 | 0.760 | 1.547 | 0.652 | 12086 | 472 |
| NHANES | 40-49 | FIB | 0.877 | 0.681 | 1.130 | 0.306 | 5916 | 722 |
| NHANES | 50-59 | FIB | 1.095 | 0.955 | 1.255 | 0.191 | 5436 | 1174 |
| NHANES | 60-69 | FIB | 1.007 | 0.898 | 1.128 | 0.907 | 5821 | 1830 |
| NHANES | >=70 | FIB | 0.930 | 0.845 | 1.024 | 0.140 | 5954 | 1760 |
| Hospital health-examination cohort | 18-39 | FIB4 | 2.011 | 1.360 | 2.960 | <0.001 | 11745 | 648 |
| Hospital health-examination cohort | 40-49 | FIB4 | 1.185 | 0.781 | 1.792 | 0.423 | 6650 | 454 |
| Hospital health-examination cohort | 50-59 | FIB4 | 1.080 | 0.933 | 1.249 | 0.299 | 6254 | 1262 |
| Hospital health-examination cohort | 60-69 | FIB4 | 1.212 | 1.038 | 1.413 | 0.015 | 4367 | 964 |
| Hospital health-examination cohort | >=70 | FIB4 | 1.182 | 0.973 | 1.433 | 0.091 | 3440 | 781 |

**Supplementary Table S5. Age-free alternative liver indices.**

| Dataset | Analysis | Term | OR | 95% CI low | 95% CI high | P value |
| --- | --- | --- | --- | --- | --- | --- |
| NHANES | NHANES: APRI per 1 SD | APRI_z | 1.091 | 1.042 | 1.142 | <0.001 |
| NHANES | NHANES: AST/ALT ratio per 1 SD | AST_ALT_ratio_z | 0.728 | 0.677 | 0.784 | <0.001 |
| Hospital health-examination cohort | Hospital health-examination cohort: APRI per 1 SD | APRI_z | 1.118 | 1.080 | 1.156 | <0.001 |
| Hospital health-examination cohort | Hospital health-examination cohort: AST/ALT ratio per 1 SD | AST_ALT_ratio_z | 1.132 | 1.093 | 1.172 | <0.001 |

**Supplementary Table S6. NHANES liver-related sensitivity analyses.**

| Sensitivity analysis | Term | OR | 95% CI low | 95% CI high | P value | N | Events |
| --- | --- | --- | --- | --- | --- | --- | --- |
| Exclude self-reported liver condition | FIB | 1.032 | 0.950 | 1.122 | 0.453 | 31382 | 4965 |
| Exclude extreme AST/ALT/platelet values | FIB | 0.854 | 0.749 | 0.972 | 0.018 | 33539 | 5548 |
| Exclude liver condition and extreme values | FIB | 0.788 | 0.689 | 0.901 | <0.001 | 29899 | 4620 |

**Supplementary Table S7. Variable-specific missingness before construction of the final analytic datasets.**

| **Dataset** | **Variable** | **Missing N** | **Missing %** |
| --- | --- | --- | --- |
| NHANES before analytic restriction | FIB-4 | 0 | 0.000 |
| NHANES before analytic restriction | Diabetes outcome | 582 | 1.455 |
| NHANES before analytic restriction | Alcohol consumption | 3295 | 8.236 |
| NHANES before analytic restriction | BMI | 547 | 1.367 |
| NHANES before analytic restriction | Sex | 0 | 0.000 |
| NHANES before analytic restriction | Race/ethnicity | 0 | 0.000 |
| NHANES before analytic restriction | Education level | 0 | 0.000 |
| NHANES before analytic restriction | Marital status | 3 | 0.007 |
| NHANES before analytic restriction | Income-to-poverty ratio | 3377 | 8.441 |
| NHANES before analytic restriction | Total cholesterol | 10 | 0.025 |
| NHANES before analytic restriction | Urinary creatinine | 623 | 1.557 |
| NHANES before analytic restriction | White blood cell count | 0 | 0.000 |
| NHANES before analytic restriction | Hemoglobin | 0 | 0.000 |
| NHANES before analytic restriction | Serum creatinine | 2 | 0.005 |
| NHANES before analytic restriction | MEC weight | 0 | 0.000 |
| NHANES before analytic restriction | Primary sampling unit | 0 | 0.000 |
| NHANES before analytic restriction | Stratum | 0 | 0.000 |
| Hospital data after import | FIB-4 | 0 | 0.000 |
| Hospital data after import | Diabetes outcome | 0 | 0.000 |
| Hospital data after import | Age | 0 | 0.000 |
| Hospital data after import | Sex | 0 | 0.000 |
| Hospital data after import | Race/ethnicity | 0 | 0.000 |
| Hospital data after import | Education level | 0 | 0.000 |
| Hospital data after import | Marital status | 0 | 0.000 |
| Hospital data after import | Smoking status | 0 | 0.000 |
| Hospital data after import | Alcohol consumption | 0 | 0.000 |
| Hospital data after import | BMI | 0 | 0.000 |
| Hospital data after import | Hypertension | 0 | 0.000 |
| Hospital data after import | Total cholesterol | 0 | 0.000 |
| Hospital data after import | Serum creatinine | 0 | 0.000 |
| Hospital data after import | White blood cell count | 0 | 0.000 |
| Hospital data after import | Hemoglobin | 0 | 0.000 |

S7 note: NHANES missingness was summarized before complete-case analytic restriction among adult participants available in the raw combined dataset. Hospital rows summarize key exposure, outcome, and Model 3 variables after data import. These summaries do not establish that excluded observations were missing completely at random or missing at random.

**Supplementary Table S8. Two-piecewise logistic regression for the nonlinear association between FIB-4 and prevalent diabetes in NHANES 2003-2018.**

| Model/segment | Adjusted OR (95% CI) | P value |
| --- | --- | --- |
| Standard logistic regression model | 1.134 (1.057, 1.217) | <0.001 |
| Inflection point | 0.737 |  |
| < 0.737 | 4.128 (2.298, 7.414) | <0.001 |
| >= 0.737 | 1.113 (1.040, 1.192) | 0.002 |
| Log-likelihood ratio test |  | <0.001 |

**Supplementary Table S9. Two-piecewise logistic regression for the nonlinear association between FIB-4 and prevalent diabetes in the hospital-based health examination population.**

| Model/segment | Adjusted OR (95% CI) | P value |
| --- | --- | --- |
| Standard logistic regression model | 1.358 (1.245, 1.480) | <0.001 |
| Inflection point | 0.745 |  |
| < 0.745 | 6.646 (4.218, 10.561) | <0.001 |
| >= 0.745 | 1.247 (1.140, 1.365) | <0.001 |
| Log-likelihood ratio test |  | <0.001 |

**Supplementary Table S10. NHANES and hospital model-specification checks and collinearity diagnostics.**

| **Panel / Analysis** | **Term** | **Estimate** | **95% CI or diagnostic** | **P value** | **N / events** |
| --- | --- | --- | --- | --- | --- |
| Panel A. Association models | FIB-4, per 1-unit increase | OR 1.358 | 1.245-1.480 | <0.001 | 32,456 / 4,109 |
| Panel A. Association models | T2 vs T1 | OR 1.511 | 1.345-1.698 | <0.001 | 32,456 / 4,109 |
| Panel A. Association models | T3 vs T1 | OR 1.761 | 1.528-2.030 | <0.001 | 32,456 / 4,109 |
| Panel B. Collinearity diagnostics | FIB-4 | GVIF 1.995 | df 1; GVIF^(1/[2 x df]) 1.412 | Not applicable | 32,456 / 4,109 |
| Panel B. Collinearity diagnostics | Continuous age | GVIF 2.062 | df 1; GVIF^(1/[2 x df]) 1.436 | Not applicable | 32,456 / 4,109 |
| Panel C. NHANES collinearity diagnostics | FIB-4 | VIF 1.280 | Fully adjusted continuous-age model | Not applicable | 35,213 / 5,958 |
| Panel C. NHANES collinearity diagnostics | Continuous age | VIF 1.520 | Fully adjusted continuous-age model | Not applicable | 35,213 / 5,958 |

Note: The hospital primary Model 3 already used continuous age; therefore, this table documents the original Model 3 specification and collinearity diagnostics rather than a separate continuous-age sensitivity analysis. The continuous FIB-4 formula was diabetes_outcome ~ FIB4 + age + sex + race + education + marital + smoking + alcohol + BMI + hypertension + TC + creatinine + WBC + HGB. The tertile model replaced FIB4 with FIB-4 tertile group; T1 was the reference category. CI, confidence interval; GVIF, generalized variance inflation factor; OR, odds ratio.

**Supplementary Table S11. Age quadratic sensitivity analyses.**

| **Dataset** | **Age model** | **Term** | **OR** | **95% CI** | **P value** |
| --- | --- | --- | --- | --- | --- |
| NHANES | age + age squared | FIB-4, per 1-unit increase | 1.054 | 0.979-1.134 | 0.158 |
| NHANES | age + age squared | T2 vs T1 | 0.880 | 0.760-1.020 | 0.088 |
| NHANES | age + age squared | T3 vs T1 | 0.957 | 0.805-1.139 | 0.619 |
| Hospital health-examination cohort | age + age squared | FIB-4, per 1-unit increase | 1.329 | 1.219-1.448 | <0.001 |
| Hospital health-examination cohort | age + age squared | T2 vs T1 | 1.270 | 1.121-1.441 | <0.001 |
| Hospital health-examination cohort | age + age squared | T3 vs T1 | 1.512 | 1.306-1.752 | <0.001 |

S11 note: Models included both linear and quadratic age terms and otherwise used the same fully adjusted covariate framework. NHANES models incorporated sampling weights, strata, and primary sampling units. T1 was the reference group for tertile analyses.

**Supplementary Figure S1. Decision-curve analyses for traditional predictors with and without FIB-4.**

**Supplementary Figure S1A. NHANES decision-curve analysis**

**Supplementary Figure S1B. Hospital health-examination cohort decision-curve analysis**

**Supplementary Figure S2. Calibration plots for traditional predictors with and without FIB-4.**

**Supplementary Figure S2A. NHANES calibration, traditional predictors**

**Supplementary Figure S2B. NHANES calibration, traditional predictors + FIB-4**

S**upplementary Figure S2C. Hospital health-examination cohort calibration, traditional predictors**

**Supplementary Figure S2D. Hospital health-examination cohort calibration, traditional predictors + FIB-4**

**Supplementary Figure S3. Exploratory subgroup analyses for the association between FIB-4 tertiles and prevalent diabetes in NHANES 2003-2018.**

**Supplementary Figure S4. Exploratory subgroup analyses for the association between FIB-4 tertiles and prevalent diabetes in the hospital-based health examination population.**
